# Association of *ADAM12* gene polymorphisms with knee osteoarthritis susceptibility

**DOI:** 10.18632/oncotarget.20772

**Published:** 2017-09-08

**Authors:** Kewei Ren, Yuan Ruan, Jilei Tang, Xuefeng Jiang, Huiqing Sun, Luming Nong, Yanqing Gu, Yuanyuan Mi

**Affiliations:** ^1^ Department of Orthopedics, The Affiliated Jiangyin Hospital of Southeast University Medical School, Jiangyin 214400, China; ^2^ Department of Minimally Invasive Spine Center, Renji Orthopedics Hospital, Shantou 515065, China; ^3^ Department of Orthopedics, Qidong People's Hospital, Nantong 226200, China; ^4^ Department of Orthopedics, Changzhou No. 2 People's Hospital, The Affiliated Hospital of Nanjing Medical University, Changzhou 213003, China; ^5^ Department of Orthopedics, Nanjing First Hospital, Nanjing Medical University, Nanjing 210006, China; ^6^ Department of Urology, Third Affiliated Hospital of Nantong University, Wuxi 214000, China

**Keywords:** a disintegrin and metalloprotease 12, knee osteoarthritis, polymorphism, risk, meta-analysis

## Abstract

Previous studies that evaluated the association between a disintegrin and metalloprotease 12 (*ADAM12*) gene polymorphisms and knee osteoarthritis (KOA) have given controversial and indefinite results. Therefore, we performed a meta-analysis to confirm this correlation. We searched the PubMed, Embase, and SinoMed databases for all papers published up to April 11, 2017. Overall, five different studies, totaling 2,353 cases and 3,668 controls, were retrieved on the basis of the search criteria for KOA susceptibility related to four polymorphisms (rs3740199, rs1278279, rs1871054, and rs1044122) in the *ADAM12* gene. Odds ratios (ORs) and 95% confidence intervals (CIs) were used to assess the strength of this association. Publication bias was assessed using Egger's and Begg's tests. The rs3740199 G/C polymorphism was found to be associated with increased KOA risk in men (e.g., CG versus GG: OR = 1.44, 95% CI = 1.02–2.04, *P* = 0.040), but not in the overall analysis and in analyses of other subgroups. Significantly increased associations were also found for the rs1871054 polymorphism (e.g., C versus T allele: OR = 1.85, 95% CI = 1.49–2.30, *P* < 0.001). However, there were no associations for the rs1278279 and rs1044122 polymorphisms. Furthermore, no obvious evidence of publication bias was detected. Our study indicated that the rs1871054 polymorphism of *ADAM12* was significantly associated with increased KOA risk.

## INTRODUCTION

Osteoarthritis (OA) is a common late-onset degenerative joint disease affecting millions of people worldwide. The prominent features of OA include progressive degradation of articular cartilage, accompanied by joint space narrowing, subchondral bone sclerosis, and osteophyte formation at the joint margin; these symptoms result in chronic joint pain and restricted motion [1]. Little is known about the etiology of OA or the relative importance of bone remodeling compared with that of cartilage degradation. In addition to age, sex, body weight, hormonal status, ethnicity, and trauma, numerous genetic factors have recently been identified as causes of OA [2, 3]. To date, several genome-wide association studies based on large sample populations have demonstrated that single nucleotide polymorphisms in various genes, such as protein kinase, cAMP-dependent regulatory type-II β (*PRKAR2B*), HMG-box transcription factor 1 (*HPB1*), component of oligomeric Golgi complex 5 (*COG5*), G protein-coupled receptor 22 (*GPR22*), growth differentiation factor 5 (*GDF5*), and a disintegrin and metalloprotease 12 (*ADAM12*) [4, 5], are associated with susceptibility to knee OA (KOA).

ADAM proteins, members of the Zn-dependent metzincin superfamily, have been shown to be associated with several complex diseases, such as rheumatoid arthritis, heart disease, Alzheimer's disease, and cancer [6, 7]. Among the 23 identified human *ADAM* genes, *ADAM12* is a candidate gene in the etiology of OA. The cellular roles of ADAM12 appear to be critical in both normal physiology and pathology. Several studies have suggested regulatory roles of human ADAM12 in bone formation, chondrocyte proliferation and maturation, and osteoclast differentiation [8–11]. Moreover, ADAM12 is upregulated in OA cartilage and multinucleated giant cells surrounding loose hip implants [12, 13]. A previous study demonstrated that expression of the ADAM12-S protein is elevated in the serum of some patients with OA and that the expression level is correlated with the grade of the disease [14]. Importantly, the expression of ADAM12 and variations of the *ADAM12* gene have previously been shown to be associated with KOA [15–20].

Considering the critical role of ADAM12 in KOA, we performed a meta-analysis of all eligible case-control studies to obtain a more accurate picture of the association of four *ADAM12* gene polymorphisms (rs3740199, rs1278279, rs1871054, and rs1044122) with KOA susceptibility.

## RESULTS

### Eligible studies

**Figure 1:**
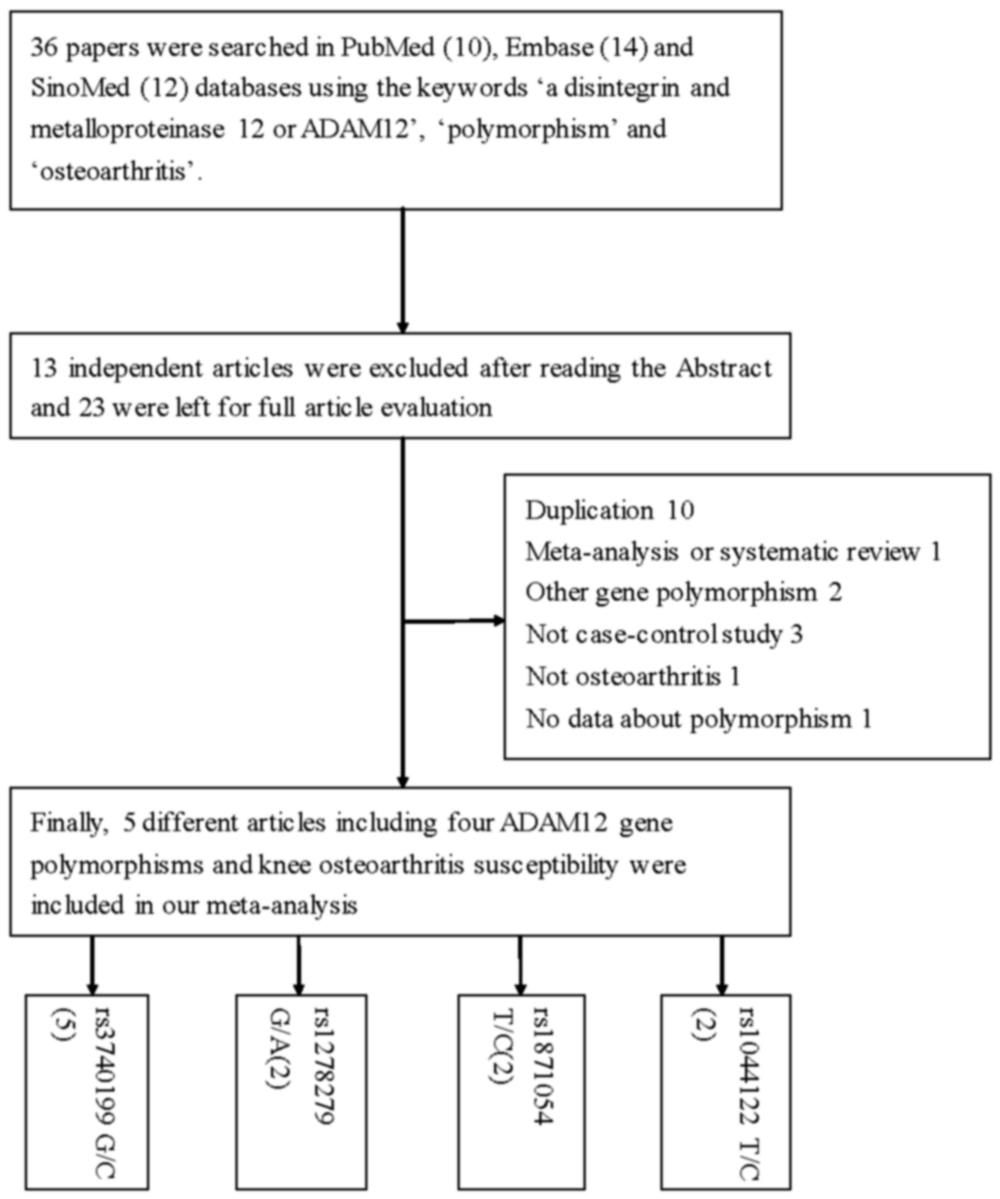
Flowchart illustrating the search strategy used to identify association studies for *ADAM12* gene polymorphisms and KOA risk

Overall, we identified five articles (11 case-control studies, four polymorphism sites) that evaluated the association of the rs3740199, rs1278279, rs1871054, and rs1044122 polymorphisms in *ADAM12* with the risk of KOA (Figure 1) [16–20]. Of these, five case-control studies, totaling 1,405 cases and 2,531 controls, were used for assessment of the rs3740199 polymorphism and KOA risk. The remaining three polymorphisms were included in two case-control studies with 316 cases and 379 controls. The diagnosis of patients with KOA was based on criteria of the American College of Rheumatology, which included primary OA with any symptoms and radiographic signs of OA according to the Kellgren-Lawrence grading system. The controls were unrelated, healthy, age-matched, ethnicity-matched individuals. The characteristics of the studies on *ADAM12* gene polymorphisms are summarized in Tables 1 and 2. Genotype distributions in the control group were consistent with Hardy-Weinberg equilibrium (HWE).

**Table 1:** Basic information for included studies of the association between *ADAM12 gene* polymorphism sites and knee osteoarthritis susceptibility

Author	Year	Origin	Ethnicity	Design	Case	Control	Case			Control				Method	NOS
							MM	MW	WW	MM	MW	WW	HWE		
**rs3740199**															
Shin	2012	Korea	Asian	PB	725	1737	147	364	214	350	863	524	0.876	TaqMan	8
Poonpet	2016	Thailand	Asian	HB	200	200	56	102	42	46	100	54	0.982	PCR–HRM	7
Kerna	2009	Estonia	Caucasian	PB	163	215	81	66	16	106	89	20	0.823	PCR-RFLP	9
Wang	2015	China	Asian	HB	164	200	36	84	44	47	102	51	0.773	iMLDR	7
Lou	2014	China	Asian	HB	153	179	32	78	43	42	93	44	0.599	real-time PCR	7
**rs1278279**															
Wang	2015	China	Asian	HB	164	200	10	62	92	15	64	121	0.119	iMLDR	7
Lou	2014	China	Asian	HB	152	179	9	59	84	13	60	106	0.274	real-time PCR	7
**rs1871054**															
Wang	2015	China	Asian	HB	164	200	76	59	29	49	99	52	0.890	iMLDR	7
Lou	2014	China	Asian	HB	152	179	69	57	26	44	88	47	0.825	real-time PCR	7
**rs1044122**															
Wang	2015	China	Asian	HB	164	200	25	88	51	37	101	62	0.712	iMLDR	7
Lou	2014	China	Asian	HB	152	179	24	81	47	31	92	56	0.517	real-time PCR	7

HWE: Hardy–Weinberg equilibrium; HB: hospital-based; PB: population-based; TB: tuberculosis; PCR-FLIP: polymerase chain reaction and restrictive fragment length polymorphism; PCR-HRM: polymerase chain reaction and high resolutionmelting; iMLDR: improved multiplex ligase improved multiplex ligase detection reaction; NOS: Newcastle-Ottawa Scale; W: wild type-allele; M: mutant-allele.

**Table 2:** The genotyping frequency of published studies on the relationship between ADAM12 rs3740199 polymorphism and knee osteoarthritis susceptibility by sex subgroup

Author	Year	Origin	Ethnicity	Sex	Case	Control	Case			Control		
							CC	CG	GG	CC	CG	GG
Shin	2012	Korea	Asian	Male	171	882	32	94	45	178	423	281
Shin	2012	Korea	Asian	Female	554	855	115	270	169	172	440	243
Poonpet	2016	Thailand	Asian	Male	53	51	19	24	10	8	25	18
Poonpet	2016	Thailand	Asian	Female	147	149	37	78	32	38	75	36
Kerna	2009	Estonia	Caucasian	Male	40	60	22	15	3	23	28	9
Kerna	2009	Estonia	Caucasian	Female	123	155	59	51	13	83	61	11

### Meta-analysis

**Figure 2:**
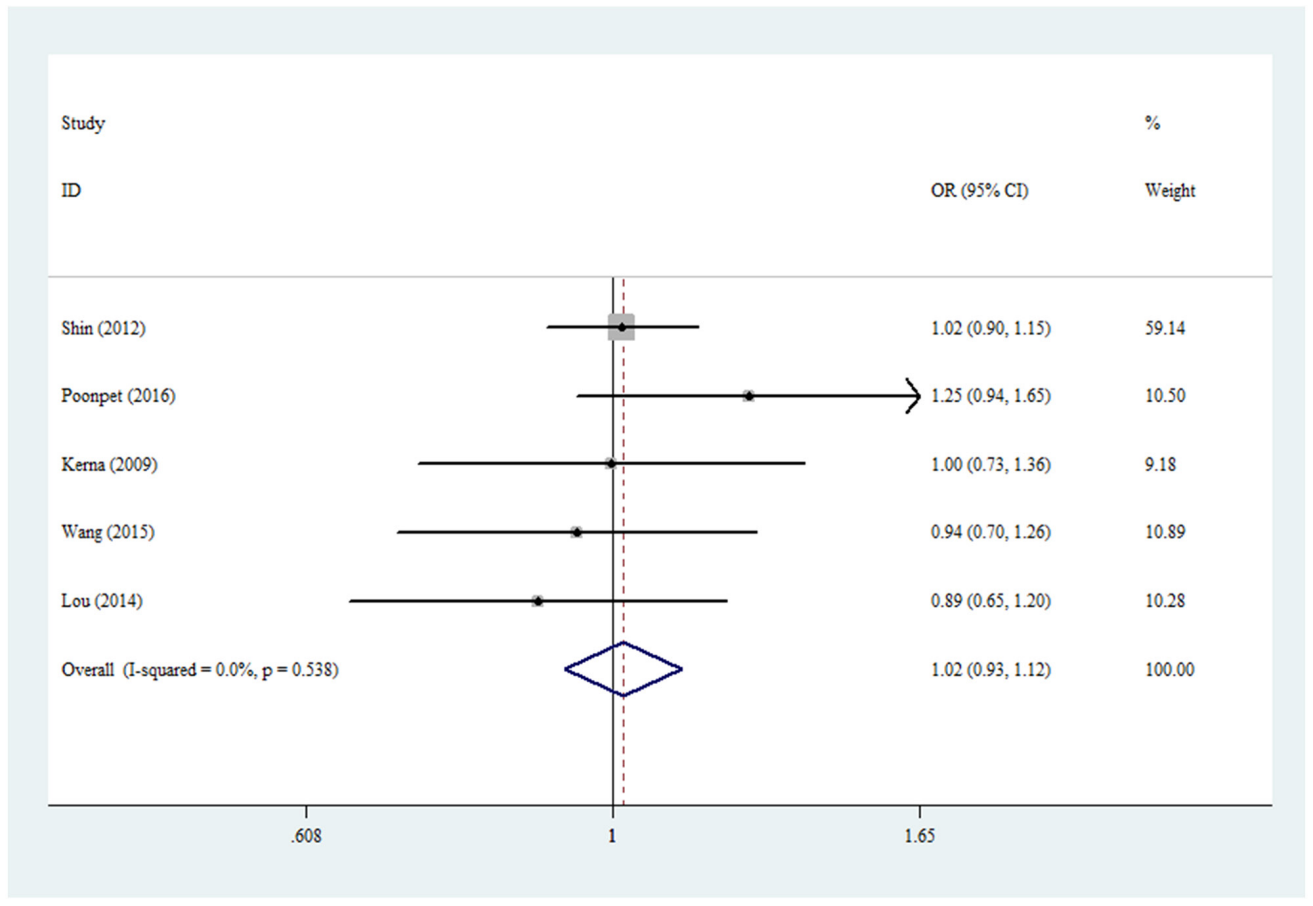
Forest plot of KOA risk associated with rs3740199 polymorphism (C-allele vs. G-allele) in the whole The squares and horizontal lines correspond to the study-specific OR and 95% CI. The area of the squares reflects the weight (inverse of the variance). The diamond represents the summary OR and 95% CI.

**Figure 3:**
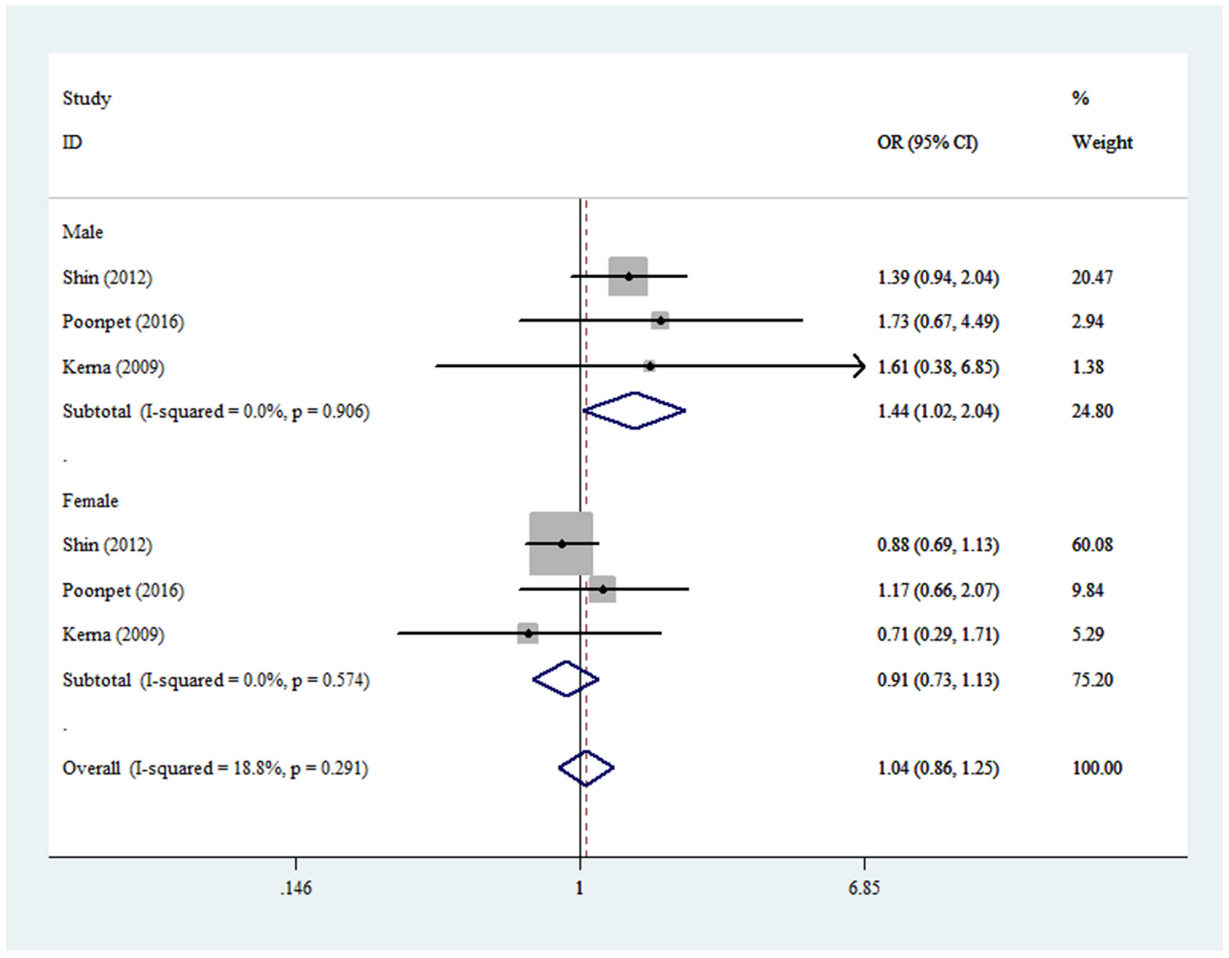
Forest plot of KOA risk associated with rs3740199 polymorphism (CG vs. GG) by sex The squares and horizontal lines correspond to the study-specific OR and 95% CI. The area of the squares reflects the weight (inverse of the variance). The diamond represents the summary OR and 95% CI.

For the rs3740199 polymorphism, no significant positive association was observed between KOA risk and the variant genotypes in all different genetic models in whole populations, including allelic contrast (odds ratio [OR] = 1.02, 95% confidence interval [CI] = 0.93–1.12, *P*_heterogeneity_ = 0.538, *P* = 0.726, fixed model, Figure 2), homozygote comparison (OR = 1.03, 95% CI = 0.85–1.25, *P*_heterogeneity_ = 0.527, *P* = 0.758, fixed model), and the dominant model (OR = 1.03, 95% CI = 0.88–1.20, *P*_heterogeneity_ = 0.631, *P* = 0.714, fixed model, Table 3A). Similarly, no associations were detected in the subgroups of ethnicity and source of control. However, despite the small number of samples included, a significant association was found for this polymorphism in men (heterozygote comparison: OR = 1.44, 95% CI = 1.02–2.04, *P*_heterogeneity_ = 0.906, *P* = 0.040, fixed model, Figure 3; dominant model: OR = 1.46, 95% CI = 1.05–2.03, *P*_heterogeneity_ = 0.420, *P* = 0.025, fixed model, Table 3A). In addition, to avoid false-positive results, we calculated the null distribution of the test statistic that adjusts for the inheritance multiple testing [21]. Results showed that the analytical *P* values were of the order of magnitude of 5.3 × 10^-6^.

**Table 3A:** Total and stratified subgroup analysis for *ADAM12 gene* polymorphism sites and knee osteoarthritis susceptibility (fixed-model)

Variables	N	Case/Control	OR(95%CI) *P*_h_ *P*	OR(95%CI) *P*_h_ *P*	OR(95%CI) *P*_h_ *P*	OR(95%CI) *P*_h_ *P*	OR(95%CI) *P*_h_ *P*
			M-allele vs. W-allele	MW vs. WW	MM vs. WW	MM+MW vs. WW	MM vs. MW+WW
**rs3740199**							
Total	5	1405/2531	1.02(0.93-1.12)0.538 0.726	1.03(0.87-1.21)0.811 0.743	1.03(0.85-1.25)0.527 0.758	1.03(0.88-1.20)0.631 0.714	1.00(0.86-1.17)0.630 0.967
Ethnicity							
Asian	4	1242/2316	1.02(0.92-1.13)0.379 0.709	1.03(0.88-1.22)0.680 0.703	1.04(0.85-1.27)0.370 0.722	1.03(0.88-1.21)0.473 0.679	1.00(0.84-1.19)0.461 0.989
Caucasian	1	163/215	-	-	-	-	-
Source of control							
HB	3	517/579	1.03(0.87-1.21)0.213 0.771	1.03(0.77-1.38)0.470 0.829	1.05(0.75-1.48)0.209 0.764	1.04(0.79-1.36)0.285 0.792	0.99(0.75-1.31)0.277 0.944
PB	2	888/1952	1.01(0.90-1.14)0.914 0.822	1.03(0.84-1.24)0.780 0.802	1.02(0.81-1.29)0.849 0.868	1.03(0.85-1.23)0.805 0.791	1.01(0.83-1.22)0.973 0.922
Sex							
Male	3	264/993	1.49(0.95-2.34)0.053 0.086	1.44(1.02-2.04)0.906 0.040	2.09(0.82-5.33)0.066 0.122	1.46(1.05-2.03)0.420 0.025	1.60(0.76-3.39)0.034 0.220
Female	3	824/1159	0.96(0.84-1.09)0.554 0.531	0.91(0.73-1.13)0.574 0.389	0.94(0.72-1.23)0.537 0.648	0.92(0.75-1.13)0.516 0.417	0.98(0.79-1.21)0.637 0.836
**rs1278279**	2	316/379	1.08(0.84-1.38)0.964 0.543	1.26(0.91-1.73)0.935 0.158	0.88(0.47-1.62)0.995 0.671	1.19(0.88-1.61)0.950 0.265	0.80(0.44-1.47)0.995 0.474
**rs1871054**	2	316/379	1.85(1.49-2.30)0.984 <0.001	1.12(0.75-1.67)0.824 0.592	2.81(1.84-4.27)0.964 <0.001	1.68(1.16-2.43)0.887 0.006	2.61(1.89-3.60)0.897 <0.001
**rs1044122**	2	316/379	0.95(0.77-1.18)0.838 0.665	1.05(0.75-1.48)0.978 0.759	0.87(0.55-1.37)0.803 0.543	1.01(0.73-1.39)0.948 0.972	0.84(0.56-1.26)0.767 0.394

*P*_h_: value of *Q*-test for heterogeneity test; *P*: *Z*-test for the statistical significance of the OR; the red mark: statistical differences by Stata software.

**Table 3B:** Total and stratified subgroup analysis for *ADAM12 gene* polymorphism sites and knee osteoarthritis susceptibility (random-model)

Variables	N	Case/Control	OR(95%CI) *P*_h_ *P*	OR(95%CI) *P*_h_ *P*	OR(95%CI) *P*_h_ *P*	OR(95%CI) *P*_h_ *P*	OR(95%CI) *P*_h_ *P*
			M-allele vs. W-allele	MW vs. WW	MM vs. WW	MM+MW vs. WW	MM vs. MW+WW
**rs3740199**							
Total	5	1405/2531	1.02(0.93-1.12)0.538 0.727	1.03(0.87-1.21)0.811 0.745	1.03(0.85-1.25)0.527 0.760	1.03(0.88-1.20)0.631 0.717	1.02(0.87-1.19)0.634 0.841
Ethnicity							
Asian	4	1242/2316	1.02(0.92-1.13)0.376 0.719	1.03(0.88-1.22)0.680 0.704	1.04(0.84-1.28)0.370 0.736	1.03(0.88-1.21)0.473 0.682	1.02(0.86-1.21)0.788 0.852
Caucasian	1	163/215	-	-	-	-	-
Source of control							
HB	3	517/579	1.02(0.83-1.26)0.213 0.845	1.03(0.77-1.38)0.470 0.832	1.04(0.68-1.60)0.209 0.846	1.03(0.76-1.41)0.285 0.828	1.03(0.78-1.37)0.428 0.831
PB	2	888/1952	1.01(0.90-1.14)0.914 0.822	1.02(0.84-1.24)0.780 0.803	1.02(0.81-1.29)0.849 0.868	1.03(0.85-1.23)0.805 0.791	1.01(0.83-1.22)0.973 0.922
Sex							
Male	3	264/993	1.25(1.02-1.53)0.053 0.030	1.44(1.02-2.04)0.906 0.039	1.50(0.99-2.28)0.066 0.056	1.46(1.05-2.03)0.420 0.025	1.24(0.89-1.72)0.034 0.212
Female	3	824/1159	0.96(0.84-1.09)0.554 0.531	0.91(0.73-1.13)0.574 0.388	0.94(0.72-1.23)0.537 0.648	0.92(0.74-1.13)0.516 0.417	0.98(0.79-1.21)0.637 0.837
**rs1278279**	2	316/379	1.08(0.84-1.38)0.964 0.543	1.26(0.91-1.73)0.935 0.158	0.88(0.47-1.62)0.995 0.671	1.19(0.88-1.61)0.950 0.265	0.80(0.44-1.47)0.995 0.474
**rs1871054**	2	316/379	1.85(1.49-2.30)0.984 0.000	1.12(0.75-1.67)0.824 0.593	2.81(1.84-4.27)0.964 0.000	1.68(1.16-2.43)0.887 0.006	2.61(1.89-3.60)0.897 0.000
**rs1044122**	2	316/379	0.95(0.77-1.18)0.838 0.665	1.05(0.75-1.48)0.978 0.759	0.87(0.55-1.37)0.803 0.543	1.01(0.73-1.39)0.948 0.972	0.84(0.56-1.26)0.767 0.395

*P*_h_: value of *Q*-test for heterogeneity test; *P*: *Z*-test for the statistical significance of the OR; the red mark: statistical differences by Stata software.

**Figure 4:**
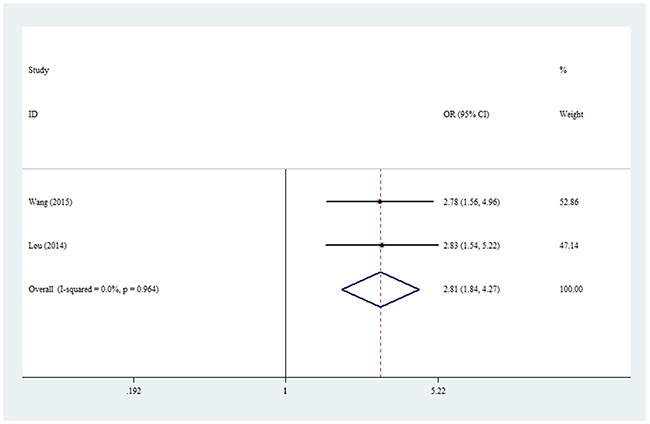
Forest plot of KOA risk associated with rs1871054 polymorphism (CC vs. TT) in the whole The squares and horizontal lines correspond to the study-specific OR and 95% CI. The area of the squares reflects the weight (inverse of the variance). The diamond represents the summary OR and 95% CI.

For the rs1871054 polymorphism, a positive correlation was found between KOA risk in the whole analysis, including allelic contrast (OR = 1.85, 95% CI = 1.49–2.30, *P*_heterogeneity_ = 0.984, *P* < 0.001, fixed model), homozygote comparison (OR = 2.81, 95% CI = 1.84–4.27, *P*_heterogeneity_ = 0.964, *P* < 0.001, Figure 4), and the dominant model (OR = 1.68, 95% CI = 1.16–2.43, *P*_heterogeneity_ = 0.887, *P* = 0.006, fixed model, Table 3A).

For the rs1278279 and rs1044122 polymorphisms, no pooled associations with KOA were found for the whole analysis (Table 3A).

Our analysis used Chi-squared and *I*^2^ to test the heterogeneity, which decides the analysis model (fixed effects model or random effects model) and is applied only one model at a time. However, the bias of this method has been pointed out by a previous study [22]. Therefore, we analyzed the opposite effects model at the same time (Table 3B), where similar results were detected in both models, which did not influence the final conclusion.

### Sensitivity analysis and publication bias

**Figure 5:**
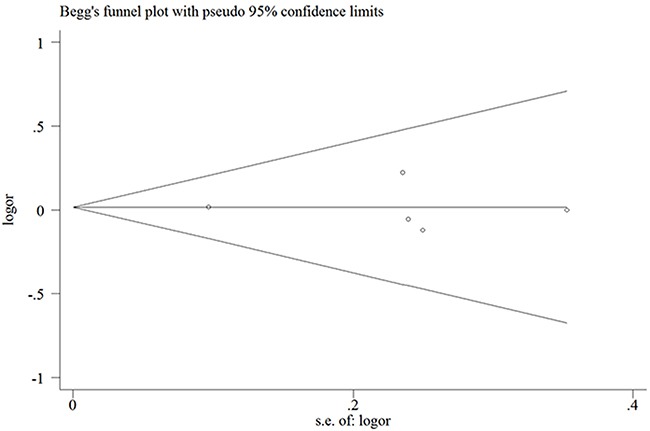
Begg's funnel plot for publication bias test in the rs3740199 polymorphism (CC+CG vs. GG) Each point represents a separate study for the indicated association. Log [OR], natural logarithm of OR. Horizontal line, mean effect size.

**Figure 6:**
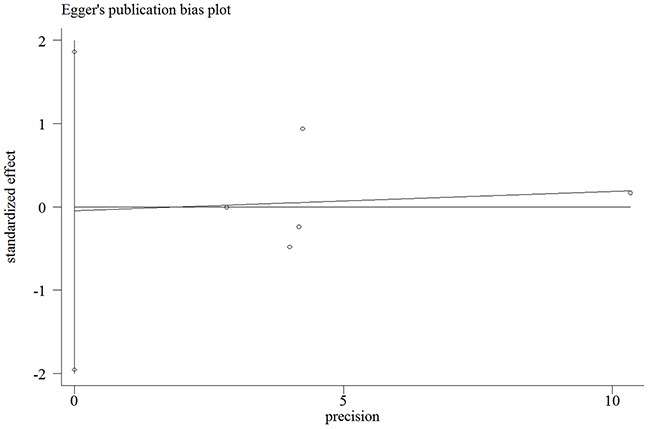
Egger's publication bias plot for the rs3740199 polymorphism (CC+CG vs. GG)

**Figure 7:**
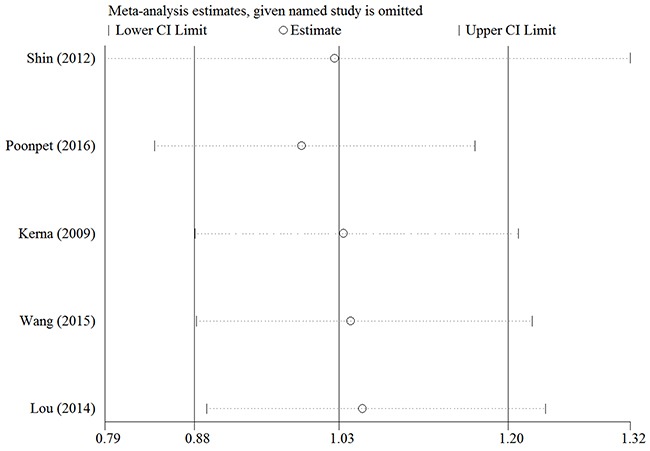
Sensitivity analysis between rs3740199 polymorphism and KOA risk (the dominant genetic model)

Egger's and Begg's tests were performed to assess for publication bias, and funnel plot symmetry was examined for the rs3740199 polymorphism (Because of insufficient degrees of freedom, the other three polymorphisms were not included and analyzed in this section). No proof of publication bias was obtained. For example, in the additive model analysis (CC+CG versus GG), we obtained values of *z* = 0.73 and *P* = 0.462 for Begg's test, and *t* = -0.08 and *P* = 0.944 for Egger's test (Figures 5 and 6, Table 4). Sensitivity analysis was performed to assess the influence of each individual study on the pooled OR by sequential removal of individual studies. The corresponding overall OR was not significantly altered with inclusion or exclusion of each study for the rs3740199 polymorphism (Figure 7).

**Table 4:** Publication bias tests (Begg's funnel plot and Egger's test for publication bias test) for *ADAM12 rs3740199* polymorphism

Egger's test						Begg's test	
Genetic type	Coefficient	Standard error	*t*	*P* value	95%CI of intercept	*z*	*P* value
**C-allele vs. G-allele**	-0.118	1.127	-0.1	0.923	(-3.705, 3.469)	0.73	0.462
**CG vs. GGCC vs. GG**	-0.043-0.057	0.5690.537	-0.08-0.11	0.9440.922	(-1.855, 1.768)(-1.767, 1.652)	0.730.73	0.4620.462
**CC+CG vs. GG**	-0.046	0.601	-0.08	0.944	(-1.958, 1.866)	0.73	0.462
**CC vs. CG+GG**	-0.101	0.775	-0.13	0.905	(-2.569, 2.367)	1.22	0.221

## DISCUSSION

Identification of gene polymorphisms related to OA susceptibility could not only make it possible to predict the disease phenotypes and construct OA prediction models based on genotype information [23], but also allow us to identify susceptible individuals for administration of preventive therapies (e.g., neuromuscular and proprioceptive training programs) in order to avoid secondary prevention measures for KOA, which typically require 10-20 min to perform and are commonly substituted for regular warm-up sessions prior to sports practice 2-3 times weekly. These programs usually involve education regarding awareness of high-risk positions. This form of prevention seems to be equally effective in all subgroups of individuals analyzed [24].

Polymorphisms in the *ADAM12* gene have been reported to be associated with KOA development and progression [11, 15, 18, 25]. Nevertheless, these conclusions are not consistent, as several other studies failed to replicate this association in other ethnicities [17, 26]. Such lack of result replication makes the application of the above-mentioned therapies difficult in the clinical setting. The differences in study results may be due to variations in genotyping and a lack of sufficient markers, differences in case ascertainment and phenotype criteria, differences in ethnicity, and the occurrence of false negatives in the replication study and false positives in the initial studies. Insufficient power related to sample size is a likely source of false positives in initial studies, which consequently tend to overestimate the genetic effects, a phenomenon called the “winner's curse” [19]. Limited power to detect genetic associations is a substantial problem in the study of the genetics of various complex diseases. Although our study was based on a relatively small population, the results enabled us to perform a meta-analysis to avoid the above-mentioned limitations.

To the best of our knowledge, this is the first meta-analysis to evaluate the association of common polymorphisms in the *ADAM12* gene with KOA susceptibility. In the overall analysis of 2,354 KOA cases and 3,668 controls, only the rs1871054 polymorphism was found to be associated with KOA risk, and individuals carrying the C allele may have a high susceptibility for the condition. Furthermore, in the sex-stratified analysis, although there was an increased association between the rs3740199 polymorphism and KOA risk in individuals carrying the CC and CG genotypes, this polymorphism site was not associated with KOA risk in the whole and subgroup analyses after using the genetic model-adjusted *P* value [22]. It is possible that different polymorphisms in the same gene may exert different effects on gene expression and function, resulting in varying KOA risks. Moreover, a single gene or a single environmental factor may not be likely to have direct effects on KOA susceptibility, and complex interactions between genetic and environmental factors may be involved in the disease development. Finally, if the number of included studies was small, false-negative results may have been found for each polymorphism.

Meta-analysis is a recognized effective method for addressing a variety of clinical questions by summarizing and reviewing published quantitative studies. However, some limitations in our meta-analysis should be mentioned. First, the number of included studies was not large enough for a comprehensive analysis, although we identified all available studies. Second, KOA risk may be modulated by gene-gene and gene-environment interactions, as well as those among different polymorphic loci in the same gene, which should be considered in further research. Third, most of the included studies were hospital-based, which may lead to lack of representation. Moreover, except for one study, all the other included studies were from Asia, which resulted in a limitation of ethnicity representation. Fourth, our meta-analysis was based on unadjusted estimates. A more precise analysis could be conducted if individual data were available, to allow for adjustment by other covariates, including age, family history, environmental factors, *ADAM12* gene expression in serum from peripheral blood, disease stage, and lifestyle. Despite the above drawbacks, our meta-analysis had three advantages: a substantial number of cases and controls were pooled from different studies, which increased the statistical power of the analysis significantly; the quality of the case-control studies included was satisfactory based on our selection criteria; and publication bias was not detected in all genetic models, suggesting that the results were relatively stable and powerful.

In summary, our present meta-analysis showed evidence of significant associations between the rs1871054 polymorphism in the *ADAM12* gene and increased KOA risk. More well-designed, large-scale studies focusing on gene-gene and gene-environment interactions are warranted to improve our understanding of the correlation between *ADAM12* gene polymorphisms and the risk of KOA development.

## MATERIALS AND METHODS

### Identification and eligibility of relevant studies

We conducted searches on the PubMed, Embase, and SinoMed databases, with the last search updated on April 11, 2017. The keywords used were “ADAM12” or “a disintegrin and metalloprotease 12,” “polymorphism” or “variant,” and “osteoarthritis” (Supplementary Table 1), without any restriction on language or publication year. A total of 36 articles were retrieved, among which five articles coincided with the inclusion criteria. We also manually screened the references of the retrieved articles and review articles.

### Inclusion and exclusion criteria

Studies that were included in our analysis had to meet all of the following criteria: (i) published studies according to the correlation between KOA and rs3740199/rs1278279/rs1871054/rs1044122 polymorphisms in the *ADAM12* gene, (ii) case-control studies, and (iii) sufficient genotype numbers (CC/CG/GG for rs3740199; AA/AG/GG for rs1278279; and CC/CT/TT for both rs1871054 and rs1044122) in both cases and controls. The following exclusion criteria were used: (i) no control population, (ii) no available number in genotyping frequency, and (iii) duplication of previous publications.

### Quality score assessment

The Newcastle-Ottawa Scale (NOS) [27] was selected to assess the quality of each study. This measure assesses aspects of methodology in observational studies related to study quality, including selection of cases, comparability of populations, and ascertainment of exposure to risks. The NOS ranges from zero (worst) to nine stars (best). Studies with a score of seven stars or greater were considered of high quality.

### Data extraction

Two of the authors independently extracted all data and ensured that they complied with the selection criteria. The following information was obtained: first author's last name, year of publication, country of origin, ethnicity, total and each genotype number in case/control group, source of controls, HWE of controls, sex data, and genotyping methods. Ethnicity was categorized as Caucasian or Asian. The control subgroups were population-based and hospital-based, and the sex subgroup was classified by male and female

### Statistical analysis

ORs with 95% CIs were used to measure the strength of associations between polymorphisms in the *ADAM12* gene and KOA. Fixed effects and random effects models were used to calculate pooled ORs. The statistical significance of the total OR was determined using *Z*-tests. Heterogeneity assumption was performed using a *χ*^2^-based *Q*-test among the studies. If the *P* value was greater than 0.10 for the *Q*-test, indicating a lack of heterogeneity among the studies, the fixed effects model (Mantel-Haenszel method) was chosen; otherwise, the random effects model (DerSimonian-Laird method) was used [28, 29]. For *ADAM12* gene polymorphisms, we investigated the association between genetic variants and KOA risk based on allelic contrast (C versus G for rs3740199; A versus G for rs1278279; and C versus T for rs1871054 and rs1044122), heterozygote comparison (CG versus GG for rs3740199; AG versus GG for rs1278279; and CT versus TT for rs1871054 and rs1044122), homozygote comparison (CC versus GG for rs3740199; AA versus GG for rs1278279; and CC versus TT for rs1871054 and rs1044122), the recessive genetic model (CC versus CG+GG for rs3740199; AA versus AG+GG for rs1278279; and CC versus CT+TT for rs1871054 and rs1044122), and the dominant genetic model (CG+CC versus GG for rs3740199; AG+AA versus GG for rs1278279; and CT+CC versus TT for rs1871054 and rs1044122). A sensitivity analysis was performed by omitting studies, one after the other, to assess the stability of results. Departure of the frequencies of the *ADAM12* gene polymorphisms from HWE was assessed by Pearson's χ^2^ test, where results of *P* < 0.05 were considered significant [30]. Publication bias was assessed by both Egger's and Begg's tests, and results of *P* < 0.05 were considered significant [31]. All statistical tests for this meta-analysis were performed using Stata software version 11.0 (StataCorp LP, College Station, TX, USA), and the power of the analysis was calculated by Power and Sample Size Calculation (http://biostat.mc.vanderbilt.edu/wiki/Main/PowerSampleSize#Windows).

### Genotyping methods

Genotyping of the polymorphisms in the *ADAM12* gene was conducted using methods described in the retrieved literature; namely, polymerase chain reaction with restriction fragment length polymorphism; polymerase chain reaction with high-resolution melting; and improved multiplex ligase detection reaction.

## SUPPLEMENTARY MATERIALS TABLES


