# Supplementary material for: Association of ADAM12 gene polymorphisms with knee osteoarthritis susceptibility
Source: Oncotarget. 2017 Sep 8;8(44):77710–21. doi: 10.18632/oncotarget.20772 (PMC5652809; doi:10.18632/oncotarget.20772)
Supplement: Supplementary file 1 [file oncotarget-08-77710-s001.pdf]

Association of *ADAM12* gene polymorphisms with knee osteoarthritis susceptibility

SUPPLEMENTARY MATERIALS

REFERENCES

1. Poonpet T, Tammachote R, Tammachote N, Kanitnate S, Honsawek S. Association between ADAM12 polymorphism and knee osteoarthritis in Thai population. Knee. 2016;23:357-361.
2. Wang L, Guo L, Tian F, Hao R, Yang T. Analysis of single nucleotide polymorphisms within ADAM12 and risk of knee osteoarthritis in a Chinese Han population. Biomed Res Int. 2015;2015:518643.
3. Lou S, Zhao Z, Qian J, Zhao K, Wang R. Association of single nucleotide polymorphisms in ADAM12 gene with susceptibility to kneeosteoarthritis: a case-control study in a Chinese Han population. Int J Clin Exp Pathol. 2014;7:5154-5159.
4. Kerna I, Kisand K, Tamm AE, Kumm J, Tamm AO. Two single-nucleotide polymorphisms in ADAM12 gene are associated with early and late radiographic knee osteoarthritis in Estonian Population. Arthritis. 2013;2013:878126.
5. El Khoury L, Posthumus M, Collins M, Handley CJ, Cook J, Raleigh SM. Polymorphic variation within the ADAMTS2, ADAMTS14, ADAMTS5, ADAM12 and TIMP2 genes and the risk of Achilles tendon pathology: a genetic association study. J Sci Med Sport. 2013;16:493-498.
6. Shin MH1, Lee SJ, Kee SJ, Song SK, Kweon SS, Park DJ, Park YW, Lee SS, Kim TJ. Genetic association analysis of GDF5 and ADAM12 for knee osteoarthritis. Joint Bone Spine. 2012;79:488-491.
7. Kerna I, Kisand K, Tamm AE, Lintrop M, Veske K, Tamm AO. Missense single nucleotide polymorphism of the ADAM12 gene is associated with radiographic knee osteoarthritis in middle-aged Estonian cohort. Osteoarthritis Cartilage. 2009;17:1093-1098.
8. Rodriguez-Lopez J, Pombo-Suarez M, Loughlin J, Tsezou A, Blanco FJ, Meulenbelt I, Slagboom PE, Valdes AM, Spector TD, Gomez-Reino JJ, Gonzalez A. Association of a nsSNP in ADAMTS14 to some osteoarthritis phenotypes. Osteoarthritis Cartilage. 2009;17:321-327.
9. Valdes AM, Van Oene M, Hart DJ, Surdulescu GL, Loughlin J, Doherty M, Spector TD. Reproducible genetic associations between candidate genes and clinical knee osteoarthritis in men and women. Arthritis Rheum. 2006;54:533-539.
10. Valdes AM, Hart DJ, Jones KA, Surdulescu G, Swarbrick P, Doyle DV, Schafer AJ, Spector TD. Association study of candidate genes for the prevalence and progression of knee osteoarthritis. Arthritis Rheum. 2004;50:2497-2507.
11. Kerna I, Kisand K, Laitinen P, Tamm A, Tamm A. Association of metallopeptidase domain 12 (ADAM12) gene polymorphisms and ADAM12 protein with the development of knee osteoarthritis. Osteoarthritis Cartilage. 2010;18.
12. Stark K, Straub RH, Blažičková S, Hengstenberg C, Rovenský J. Genetics in neuroendocrine immunology: implications for rheumatoid arthritis and osteoarthritis. Ann N Y Acad Sci. 2010;1193.
13. Limer KL, Tosh K, Bujac SR, McConnell R, Doherty S, Nyberg F, Zhang W, Doherty M, Muir KR, Maciewicz RA. Attempt to replicate published genetic associations in a large, well-defined osteoarthritis case-control population (the GOAL study). Osteoarthritis Cartilage. 2009;17:782-789.
14. Oliver JE, Silman AJ. What epidemiology has told us about risk factors and aetiopathogenesis in rheumatic diseases. Arthritis Res Ther. 2009;11:223.
15. Lai YH. To study the effect of metalloproteinase ADAMTS-7 in soft foot bone development and the pathogenesis of osteoarthritis. 2011. Shandong University. A doctor’s degree thesis.
16. Xia P, Li XP, Lin Q, Chen K, Shen SH, Ren SS, Gao MX. The effects of low intensity pulsed ultrasound on expression of mechanochemical transduction pathway-related protein of integrin-focal adhesion kinase-mitogen-activated protein kinases in the chondrocytes of rabbits with knee osteoarthritis. Chin J Phys Med Rehabil. 2014;36.
17. Bai XH. Functional study of ADAMTS-7 and ADAMTS-12 inhibiting cartilage differentiation. 2009. Shandong University. A doctor’s degree thesis.
18. Lin QS. The role of interleukin -17 in the pathogenesis of intervertebral disc degeneration. 2011. The Second Military Medical University. A doctor’s degree thesis.
19. Huang M. The experimental study of the role of ADAMTSs in intervertebral disc degeneration. The Fourth Military Medical University. 2011. A doctor’s degree thesis.
20. Huang YJ. Living protection strategy of Chongqing traditional style area based on historical continuity -- Taking eighteen ladder traditional style area in Chongqing as an example. Chongqing University. 2016. A master’s degree thesis.
21. Wang R. Association between single nucleotide polymorphisms in the integrin metalloprotease 12 gene and susceptibility to knee osteoarthritis: a case-control study in

Chinese Han population. 2015 annual academic meeting of Othopedics in Zhejiang. Hangzhou, Zhenjiang, China.

22. Hui J. Association of eight Alzheimer disease susceptibility genes in northern China. Ningxia Medical University.2014. A master's degree thesis.
23. Meng XM, Yu SF. Expression of a disintegrin-like and metalloproteinase protein 8 and 12 in the giant cell lesions of jaw. Chin J Stomatol. 2004;39.
24. Meng XM, Yu SF, Lu M, Zheng J, Han ZH. Expression of macrophage inflammatory protein-1α, a disintegrin-like and metalloproteinase 8 and 12, and CD68 protein in giant cell lesions of jaw and giant cell tumors of long bone. Chin J Pathol. 2005;34.
25. Tian BL. Study on the relationship between ADAM12 expression and multinucleated giant cell formation in giant cell tumor of bone. Zhongshan University. 2002. A master's degree thesis.
26. Zhu LN, Ma RX. Correlation of aggressive fibromatosis genes. Chin J Pediatr Surg. 2016;37.

Supplementary Table 1: Search strategies and detailed records

Relevant text of ADAM12	Relevant text of knee osteoarthritis
1. A disintegrin and metalloprotease	15. Arthritis, Degenerative
2. ADAM12 gene	16. Osteoarthrosis
3. ADAM	17. Osteoarthrosis Deformans
4. Matrix metalloproteinases	18. osteoarthritis
5. Proteolytic enzymes	19. OA
6. Polymorphism	20. KOA
7. Variant	21. Degenerative joint disease
8. SNP	22. The most common form of arthritis, especially in older persons
9. Single nucleotide polymorphisms	23. 15 or 16 or 17 or 18 or 19 or 20 or 21 or 22
10. rs3740199	**Combined (Final strategy)**
11. rs1278279	24. 14 and 23
12. rs1871054	
13. rs1044122	
14. ((1 or 2 or 3 or 4 or 5) and (6 or 7 or 8 or 9)) or 10 or 11 or 12 or 13	

Web sites and uniform resource locator:
PUBMED: http://www.ncbi.nlm.nih.gov/pubmed
EMBASE: https://www.embase.com
SinoMed: (WanFang: http://s.wanfangdata.com.cn/; CNKI: http://kns.cnki.net/)

Records from PUBMED [1–10]:

Unrelated records (wrong outcome [4,5,8,9,10])
Included studies [1,2,3,6,7]

Records from EMBASE [1-10, 11-14]:

Duplicated records [1–10]
Unrelated records (wrong outcome [11–14])

Records from SinoMed [15–26]:

Unrelated records (wrong outcome 15-26])
